# P-697. Increases in Ages and Bacterial Coinfection in Hospitalized Children with Respiratory Syncytial Virus Infection in Taiwan:2019- 2023

**DOI:** 10.1093/ofid/ofae631.893

**Published:** 2025-01-29

**Authors:** Jia Yi Gui, En-Wei Hsing, Chong-Wei Huang, Kuo Chien Tsao, Yu-Chia Hsieh, Chih-Ho Chen

**Affiliations:** Department of Pediatrics, Chang Gung Memorial Hospital, Taoyuan, Taoyuan, Taiwan (Republic of China); Chang Gung University, Taoyuan, Taoyuan, Taiwan; Keelung Chang Gung Memorial Hospital, department of Pediatric / Attending, Taoyuan, Taoyuan, Taiwan (Republic of China); Research Center for Emerging Viral Infections, Chang Gung University, Taoyuan, Taiwan, Guishan, Taoyuan, Taiwan; Linkou Chang Gung Memorial Hospital, Taipei, Taipei, Taiwan; Kaohsiung Chang Gung Memorial Hospital, Kaohsiung, Kaohsiung, Taiwan

## Abstract

**Background:**

Efforts to expand RSV epidemiological data are crucial, especially considering upcoming interventions such as long-acting monoclonal RSV-neutralizing antibodies and maternal vaccines for RSV prevention, which have the potential to alter the landscape.

**Methods:**

A retrospective study was conducted at Chang Gung Memorial Hospital, analyzing children hospitalized with RSV infection before, during and after the COVID-19 pandemics from 2019 to 2023. Clinical data were collected and stratified by year for analysis. Plasma microbial free-DNA sequencing (mcfDNA) was performed on selected cases with C-reactive protein > 100 mg/L to enhance diagnostic yields.

**Results:**

A total of 997 children hospitalized for RSV infection was enrolled. In 2019, 2022, and 2023, the incidence of RSV-related hospitalization among children aged 0-4 years remained relatively stable, ranging between 600-700 per 100,000. However, in 2020 during a strict national nonpharmaceutical intervention, there was a notable spike, doubling to reach a peak, followed by an 80% decrease in 2021. In age subgroup analyses comparing 2022 and 2023 to 2019, the incidence of RSV-related hospitalization among children aged < 2 years gradually decreased from 1576 per 100,000 in 2019 to 1212 per 100,000 in 2022 (P=0.03) and 1262 per 100,000 (P=0.07) in 2023. Conversely, the incidence of RSV-related hospitalization among children aged 2-4 years significantly increased from 124 per 100,000 in 2019 to 347 per 100,000 in 2022 (P< 0.001) and 419 per 100,000 in 2023 (P< 0.001). Independent risk factors for CRP >100 mg/L in RSV cases were year 2023 (aOR:4.03, P=0.008) and age 2-4 years (aOR:3.33, P< 0.001). In 2023, among 23 children with CRP >100 mg/L, *Hemophilus influenzae* was identified in two children receiving bronchoalveolar lavage, and in 73% (11/15) of samples underwent plasma mcfDNA.

**Conclusion:**

Following the COVID-19 pandemic, there was a proportional shift in RSV cases towards children over 2 years of age. Increase in age exhibited a higher rate of bacterial infections, particularly with *Haemophilus influenzae*. These findings offer critical implication for RSV prevention strategies and treatment of community-acquired pneumonia.

**Disclosures:**

**All Authors**: No reported disclosures

